# Capacity as “virtual stock” in ecosystem services accounting

**DOI:** 10.1016/j.ecolind.2018.10.066

**Published:** 2019-03

**Authors:** Alessandra La Notte, Sara Vallecillo, Joachim Maes

**Affiliations:** European Commission Joint Research Centre, Directorate D – Sustainable Resources, Via E.Fermi 2749, 21027 Ispra, VA, Italy

**Keywords:** Capacity, Ecosystem services, Ecosystem accounting, Environmental accounting

## Abstract

Interest in ecosystem services accounting is growing exponentially. There are many un-solved issues that need to be addressed, the notion of capacity is among them. International guidelines suggest that capacity should constitute the link between the ecosystem assets accounts and the ecosystem services accounts. In order to address this issue, the authors use Supply and Use table for ecosystem services to show the relationship between (i) ecosystem assets and (ii) capacity, intended as “virtual stock” of individual ecosystem services. It is in fact important to distinguish ecosystem assets from capacity as “virtual stock”: a comparison between asset accounts for natural resources and capacity is presented to clarify this difference. This novel approach is described together with its implications in order to feed further developments and discussion.

## Highlights

• The capacity account should link ecosystem condition with ecosystem services accounts.
• Asset accounts for ecosystems differs from asset accounts for natural resources.
• Capacity is calculated for individual ecosystem services and not for ecosystems.
• Capacity represents a virtual stock providing annual flows of ecosystem services.
• Capacity as “virtual stock” differs from ecosystem assets.

## Graphical abstract

**Figure:**
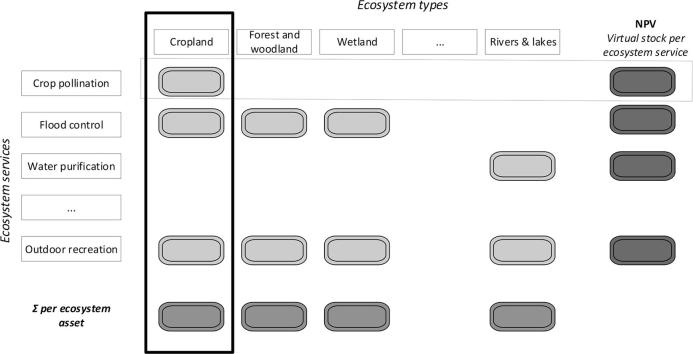

## 1 Introduction

Interest in ecosystem services accounting is growing exponentially. The integration of ecosystem and economic accounts would allow to mainstream information on ecosystem services into decision-making from strategic planning at national scale to management purposes at subnational scales. Processing ecosystem services information can be directly integrated into already existing economic tools (such as extended input-output tables, general equilibrium models); they can also be combined with ad hoc sectoral policies according to specific features and characteristics (such as policy targets established by specific regulations).

Not only international institutions are proposing frameworks and guidelines (United Nations, 2017, United Nations et al., 2014b), but also the scientific community is putting a considerable effort in conceptual and empirical work to deal with this complex issue (Hein et al., 2016, La Notte et al., 2017c, Obst, 2015). Currently the most widely applied framework is the System of integrated Environmental and Economic Accounting – Experimental Ecosystem Accounts (SEEA EEA). The SEEA EEA has been developed under the supervision of the United Nation Statistical Division. Updates, applications and new proposals are regularly discussed by the London Group, 1 an expert group, which aims to advance knowledge, implementation and applications on the SEEA. The World Bank recalls the SEEA framework in its Wealth Accounting and the Valuation of Ecosystem Services (WAVES) initiative^2^ that is a global partnership aiming at ensuring the mainstream of natural resources in development planning and national economic accounts. WAVES supports countries to adopt and implement accounts, to develop approaches to ecosystem accounting methodology, to establish a global platform for training and knowledge sharing, and to build international consensus around natural capital accounting. The European Commission aims to apply the SEEA framework in its Knowledge Innovation Project Integrated system for Natural Capital Accounts (KIP INCA) initiative. 3 The objective of INCA is to build biophysical and monetary accounts at European Union (EU) level to respond to EU policies. The steps leading to the EU account are: to expand the database available for filling out the accounting forms, to provide a series of tests, experiments and demonstrations on natural capital in Europe, and to formulate guidelines leading. Within this project, the Joint Research Centre of the European Commission is specifically elaborating supply and use tables of ecosystem services in physical and monetary terms. SEEA EEA is also promoted by other international NGOs such as Conservation International^4^ (CI) and worldwide initiatives such as The Economics of Ecosystem and Biodiversity^5^ (TEEB).

There are many unsolved issues that will be addressed by the SEEA EEA revision (Hein et al., 2016, United Nations, 2017). Among those issues, the notion of capacity is still under debate. The purpose of this short note is to propose a consistent frame for capacity, able to combine the notion of ecosystem asset with ecosystem services in a coherent way from both an ecological and accounting perspective.

## 2 The accounting structure for ecosystem services and capacity

**Fig. 1:**
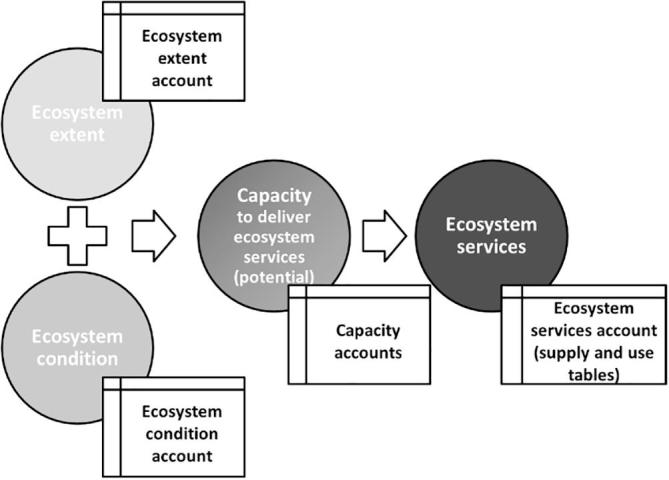
Components of a natural capital accounting framework (source: (Maes et al., 2018)). Ecosystem extent and condition determine, among other factors, the ecosystem capacity to deliver service that will ultimately determine the ecosystem services.

The SEEA EEA guidelines (European Commission and European Environment Agency, 2016, United Nations, 2017, United Nations et al., 2014b) suggest that capacity constitutes the link between the ecosystem assets accounts and the ecosystem services accounts (Fig. 1).

Extent and condition directly affect the capacity of ecosystems to provide services even if this relationship is often not linear: sustainable management practices are positively related to ecosystem condition but intensive use has a negative impact on ecosystem condition and will result in ecosystem degradation (Maes et al., 2018). Environmental policies aims to reduce pressure on the environment and this would likely enhance ecosystem condition.

The latest version of the SEEA EEA Technical Recommendations (United Nations, 2017) states that capacity is not measured in terms of an account at this stage. In the literature, we find capacity defined sometimes as a flow (Hein et al., 2016, Schröter et al., 2014) and sometimes as a stock (La Notte et al., 2017b, Villamagna et al., 2013). This is not a trivial issue: this definition will indeed affect the whole ecosystem services accounting structure, whose procedure is now explained.

The applications undertaken so within KIP INCA (La Notte et al., 2017c, Vallecillo et al., 2018) consider the following steps:

• biophysical assessment of ecosystem services, that can be undertaken according to a tiered approach (ranging from indicators to biophysical modelling). According to scale and the geographical location, the biophysical assessment will be fed with specific datasets and calibrated accordingly. What matters for resolution is the adopted minimum reference unit (e.g. cell of a grid 1 km × 1 km, river catchment, Local Administrative Unit, etc.);
• translation of the biophysical outcome in monetary terms: the biophysical assessment leads any occurring change, so that monetary units will be specular to physical units. As long as the features and meaning of the biophysical assessment stay intact, appropriate valuation techniques can be selected, making sure to preserve the consistency with the SNA (i.e. exchange values or transaction prices);
• accounting in physical and monetary terms: the structure proposed in the SEEA EEA is kept consistent with the SNA for both asset and ecosystem services flow accounts. National accounts are provided at national scale, but can also be compiled at subnational scales from regional to municipal. By adopting a minimum reference unit with a high resolution (and in case appropriate modelling with appropriate parameters) allows to compile accounts at local scale.

**Fig. 2:**
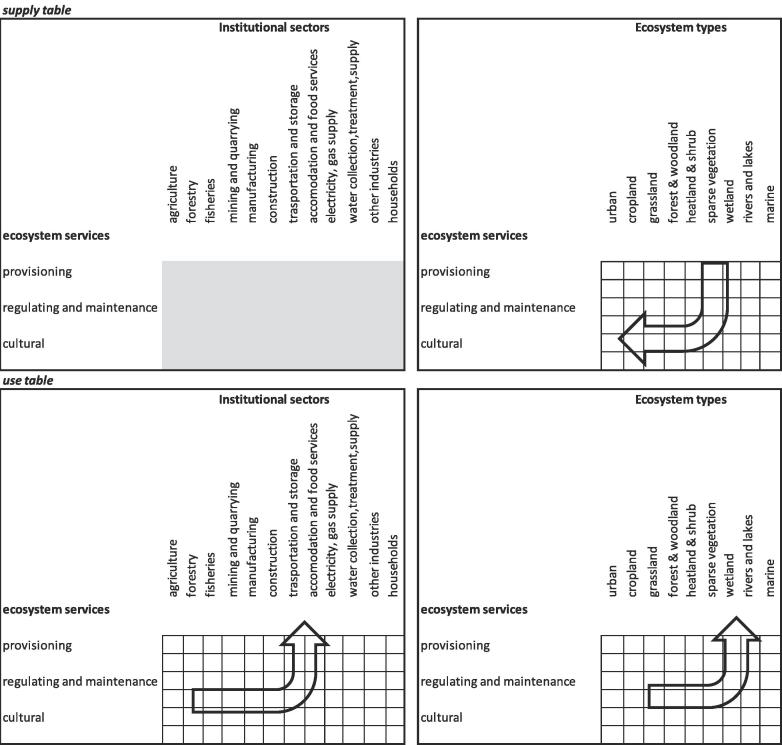
General presentation of supply and use table. Institutional sectors are organized according to NACE rev.2 classification; ecosystem types are organized according to a combined MAES and CORINE classification. According to the specificity of the scale subgroups that are more detailed could be fit within the main classification system.

Accounting for ecosystem services requires specifically Supply and Use Tables (SUT) where the demand, represented by economic sectors and households, interacts with the supply of services from ecosystem assets, organized in ecosystem types according to the SEEA EEA frame. The actual flow of ecosystem services is here reported. Through the supply table, it is possible to track from which ecosystem assets each of the services does flow. Through the use table it is possible to track to which economic sectors and/or households each of the services does flow (Fig. 2). Usually the accounting period is one year, but for some ecosystem services (especially those where the driver of change is land use) it might be sufficient to consider 5–10 years.

**Fig. 3:**
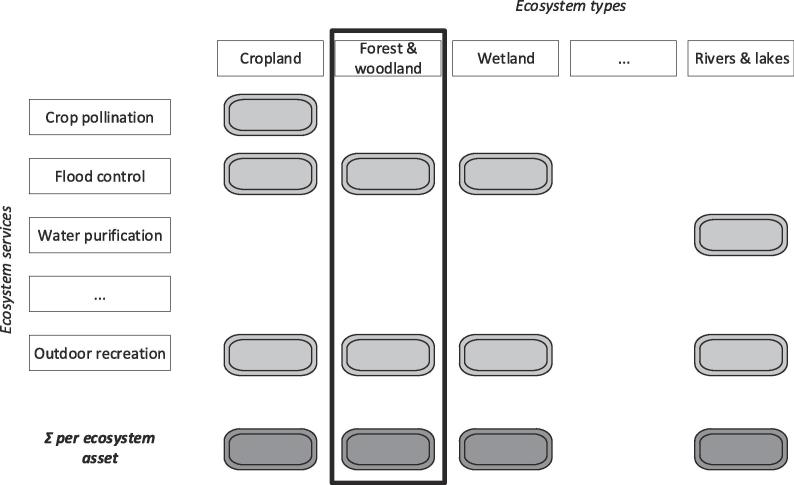
Relationship between ecosystem types and ecosystem services in the supply table – ecosystem assets.

In building the supply table, the flow of each ecosystem service is allocated to the specific ecosystem asset it comes from. The allocation itself depends on the specific service being assessed and the assumptions adopted for the biophysical assessment, e.g., in the case of crop-pollination the ecosystem assets belong to croplands because is the ecosystem type where the use of the service takes places (i.e. where is needed). In the case of outdoor recreation almost all (with few exceptions), ecosystem assets are involved. For water purification, the assumptions of the modelling enable the allocation of the service flow only to inland waters. Once a representative number of ecosystem services is calculated and translated in a common monetary unit, it is possible to sum those flows and estimate the value of each ecosystem type (Fig. 3).

**Fig. 4:**
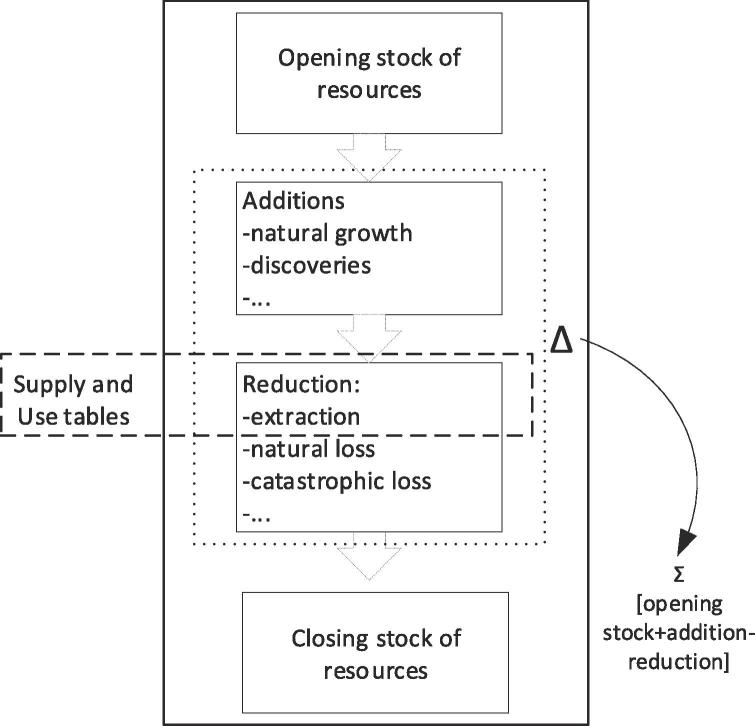
Asset accounts for natural resources (source: adapted and modified from (United Nations et al., 2014a)). This simplified visualization shows the changes (Δ) that could affect the opening stock during the accounting period. The closing stock will be the sum of opening stock plus addition and minus reduction.

Ecosystems can be considered assets. Asset accounts are filled in for natural resources in the SEEA Central Framework (SEEA CF) and in the SEEA Agriculture, Forestry and Fisheries (SEEA AFF). The way asset accounts are filled for natural resources and for ecosystems may not be the same. In SEEA CF and in SEEA AFF, in fact, each natural asset provides a single flow, e.g. in timber asset accounts the flow is reported mainly as the annual increment of wood biomass (+) and the annual removal of wood (−). Fig. 4 offers a simplified visualization of how asset accounts work in SEEA CF and SEEA AFF.

In the SEEA-EEA, a single ecosystem asset can provide a variety of services, e.g. forest provide not only wood biomass but also carbon sequestration, flood protection, erosion control, soil decontamination, outdoor recreation and pollination. Capacity is commonly defined as the long-term ability of different ecosystem types to provide different ecosystem services (Burkhard and Maes, 2017, Potschin et al., 2016). In order to account for capacity we should thus focus on individual ecosystem services (Hein et al., 2016) rather than ecosystem assets because on the one hand different ecosystem types can provide (all together) a single service, and on the other hand the a single ecosystem type can provide several ecosystem services.

**Fig. 5:**
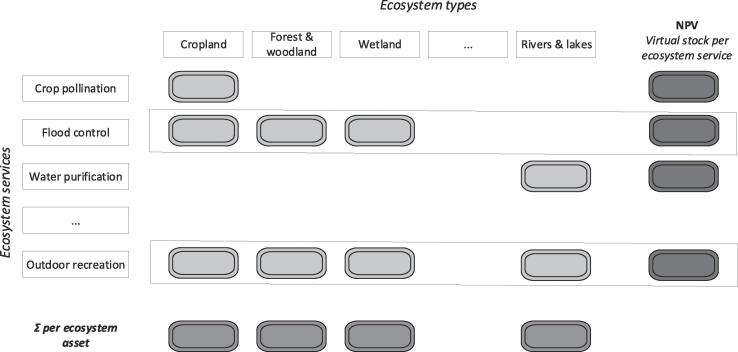
Relationship between ecosystem types and ecosystem services in the supply table– capacity as virtual stock.

The assessment of capacity in monetary terms is strictly related to its assessment in biophysical terms. In this paper, we only focus on the monetary assessment of the capacity, following the guidelines presented in the SEEA EEA TR (ref. chapter 7 in United Nations, 2017). The SEEA EEA guidelines suggest to calculate capacity as the Net Present Value (NPV) of the annual flow of the ecosystem services. The NPV is the value at present of what will be provided today and for the years to come (lifetime). The NPV approximates, in monetary terms, the long-term ability of ecosystem assets to provide each individual ecosystem service, which can be considered as a “stock” (Fig. 5). The term “stock” is here used just to highlight how, in this case, capacity (as NVP per ecosystem service) is not a synonym of asset.

Fig. 5 needs to be carefully interpreted: the last column describes the NPV for each specific ES, i.e. it is a function that depends on the ecosystem service flow expressed in monetary terms; while the last row (Fig. 3, Fig. 5) is the sum of the annual flows of different ecosystem services per ecosystem type, expressed for the same spatial extent.

The notion of ecosystem asset, which corresponds to each ecosystem type, is separated from the notion of capacity, which refers to individual ecosystem services. The latter is an unconventional “stock”, meant to simplify the complexity embedded in ecosystem services measurement; which can only be measured in monetary terms. This stock can be called “virtual” because we deal with the ability to keep on generating an ecological process over time (expressed in monetary terms) and not with something that can be physically accumulated. The structure shown in Fig. 3, Fig. 5 is meant to be consistent with the frame of external satellite accounts on which the SNA and the SEEA are based.

**Fig. 6:**
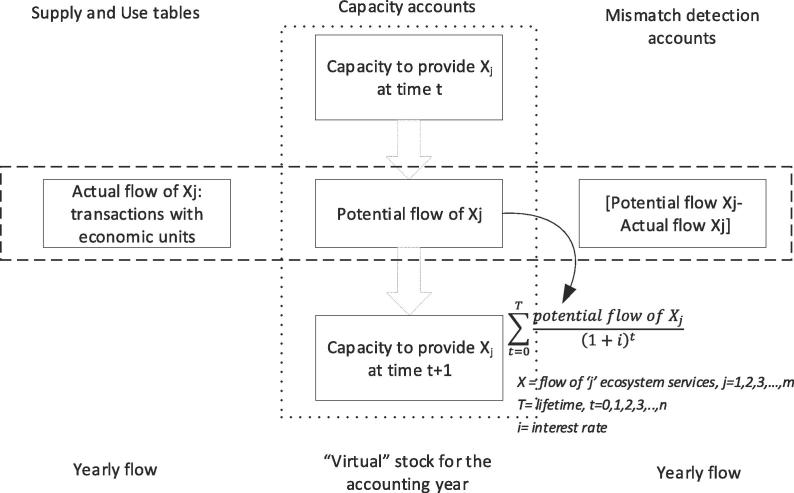
Capacity as “virtual stock” account. This simplified visualization shows the linkage among capacity as NPV, supply and use tables and a mismatch account that (when it is negative) can measure overuse. It also highlights how the capacity is calculated from potential flow. This feature applies to those ecosystem services where regeneration and absorption rates matter.

Fig. 6 shows that capacity at time t represents the opening stock of the 'long term available ecosystem service' in monetary terms. There are some ecosystem services where regeneration and absorption rates can be impacted by unsustainable human use. If a sustainability threshold can be established, it becomes possible to calculate what we can call “potential flow” (or sustainable flow). If the actual flow of the service (the use) is equal or below the potential flow, then the capacity to provide the same (or enhanced) amount of ecosystem service is guaranteed. If the actual flow is higher than the potential flow, then a “mismatch accounts” would record overuse that (in the medium/long term) will eventually lead to degradation. The capacity to provide the ecosystem service for the following year should thus be calculated by using the potential flow. Actually, the capacity calculated using the actual flow in cases of overuse (unsustainable use) would be higher than the capacity calculated using the potential flow, that considers sustainability criteria when assessing the services delivered by the ecosystems. This latter application of capacity would neglect the foundation of ecosystem accounting: overuse of the service and the subsequent ecosystem degradation should in fact be reflected in a decline in capacity.

The potential flow shown in Fig. 6 is not going to be part of the conventional supply and use tables, which will only report the actual flow (i.e. transaction with economic actors). Potential flows can be used to fill in complementary tables for all those ecosystem services characterized by regeneration and absorption rates, such as provisioning services or sink-related services. However, when there is a mismatch between potential and actual flow, this difference needs to be measured and future capacity (opening stock at time t+1) should be calculated from the potential flow.

One important feature to be kept in mind by practitioners is that in Fig. 4 (natural resources) we deal with mass (e.g. water, subsoil assets) and biomass (e.g. crops, timber, fisheries), and in Fig. 6 (capacity as “virtual stock” of ecosystem services) we deal with service flows. In order to further clarify this difference, we briefly recall the categories of system ecology (Jørgensen, 2012). At the basis of ecological systems there are three notions: biomass, interaction and information. Biomass is simply the biological material derived from living or dead organisms. Interactions and information are the relationships among mass (abiotic components) and biomass (biotic components). A higher degree of complexity characterizes ecosystem services (interaction and information) that take place within ecological networks. They cannot be treated as mass and biomass because they are indeed the relationship between mass (abiotic components) and biomass (biotic components) (La Notte et al., 2017a). Based on that, intra and inter ecosystem flows (among which biodiversity and its loss) do play a role in assessing the flow of ecosystem service, and thus its decreasing (or increasing) capacity as result of human action (Fig. 6).

In ecosystem services accounting the amount of natural resources should not be used as proxy for the service flow as it is; even in simplified procedures adopted for provisioning services, the service as ecosystem contribution (SEEA EEA) should be separated from the actual resource generated (SEEA CF) in order to avoid misleading information (Pèrez-Soba et al., 2015).

Any practitioners should keep in mind that any number reported in the supply and use table (aggregated at sub-regional, regional, national, continental scales) is the outcome of a biophysical assessment working together with a monetary valuation model. The biophysical assessment will likely be a spatially explicit ecological model: this implies that any crucial functional relationship will be spatially represented. Functional relationships affecting regeneration and absorption rates will change potential and actual flows that will change the NPV of capacity.

## 3 Discussion

This “virtual stock” frame for capacity has some implications. Firstly, capacity is calculated for individual ecosystem services and not for ecosystems. 6 The advantage lies in a more transparent approach: by tackling ecosystem services individually, practitioners know exactly what they are assessing and how (i.e. proxies and limitations). This kind of analysis enable to identify ecosystem types and/or services under particular threats. The drawback lies in the lack of direct integration/interaction among all the ecosystem services: any trend of synergies and trade-offs can only be checked ex-post because different models will likely be used for different ecosystem services (not just one integrated model for the whole ecosystem). In other words, the trade-offs between different baskets of ecosystem services derived from ecosystem types can contribute to the generation of a variety of goods and services upon which people depend: each ecosystem asset generates in fact a number of different ecosystem services that are subject to complex, non-linear dynamics involving negative or positive feedback loops.

Secondly, the fact that the notion of capacity is based on the measurement of the potential flow allows establishing a link with condition accounts to be compiled for ecosystems. Condition accounts would in fact deal with the state of the ecosystem and contain indicators related to: physical and biological condition (e.g. soil fertility, water quality, vegetation health), ecological processes (e.g. net primary production) and presence of species (e.g. species richness, endangered species, conservation flagship species). Most of these features are also employed in the biophysical models used to assess the potential flows of corresponding ecosystem services. For example: soil fertility is a critical variable for the ecosystem service “protection against soil erosion”; water quality (i.e. impact of pollutants) is a critical variable for the ecosystem service ”water purification“; net primary production is a critical variable for the ecosystem service ”timber provision“ (i.e. increment of wood biomass); species richness is a critical variable for the ecosystem service ”habitat maintenance“. Once the relation is clearly identified, it will be possible to establish a linkage between ecosystem condition accounts and ecosystem services supply and use tables and eventually the contribution of ecosystems to the monetary values. Based on the assumption that all biophysical models used for ecosystem services accounting are spatially explicit, we are aware that, for each ecosystem service and each ecosystem type, applications are needed to demonstrate how the linkage with condition accounts (in principle feasible in accounting terms) can be put in place.

Thirdly, the fact that the notion of capacity is based on the measurement of the potential flow allows to systematically assessing degradation within the accounting system and thus monitoring sustainability changes in natural capital. The overuse of the service flow can be measured by confronting the potential flow and the actual flow (ref. Fig. 6). The mismatch between the two flows can occur for those typologies of ecosystem services where regeneration and absorption rates matter: (i) regeneration rates affect provisioning services such as plant biomass increment and fish biomass maintenance, (ii) absorption rates affect sink-related services such as soil decontamination, water purification and air filtration. Human use that overexploits regeneration and absorption rates is unstainable and generates over time degradation. Concepts of resilience, thresholds and irreversibility should in fact be an important component of ecosystem accounting, especially for those ecosystems dominated by complex dynamics (such as temperate and tropical forests, rangelands, estuaries, and coral reefs).

Fourthly, capacity is comparable to the notion of stock and not to the notion of flow. Actual flow can be higher (unsustainable), equal or lower (sustainable) than potential flow; actual flow cannot be higher than capacity, unless the ecosystem service is eradicated (due for example to clear cut or land use conversion [from forest to grassland] or due to the discharge of a massive load of pollutants that kill biological organisms). If this conceptual definition of capacity is adopted, terminology should be used accordingly.

Finally, capacity should neither be confused nor mixed with ecosystem assets, that are measured through extent and condition accounts (Fig. 1). Being related to ecosystem services (Fig. 4), the measurement of capacity starts from supply and use tables and expands from them (Fig. 5). When a representative number of ecosystem services are available for the same ecosystem asset/type, then, their NPV could be summed up and an ecosystem asset capacity account can be proposed. The whole accounting frame should at the end of the day be internally consistent and coherent with the national accounts (Obst, 2015, United Nations, 2017, United Nations et al., 2014b). This would assure the integration of ecosystem services accounts into the economic accounts: the linkage lies in the allocation of services to the users (ref. use table in Fig. 2) that can be economic sectors and households. By moving from ecosystem assets to ecosystem services and to economic accounts, it is possible to describe chains connecting ecological interaction to services and subsequently to benefits; these chains may be particularly important for assessing the ecosystem-wide implications of specific decisions, and *vice versa*.

The methodological separation between accounts for ecosystem service capacity and ecosystem asset condition ecosystem services capacity accounts and ecosystem asset condition accounts is functional. The two sets of accounts serve in fact different purposes. To address policy questions concerning ecosystems as a whole, it makes sense to refer to condition accounts: e.g. monitoring conservation issues does require a holistic perspective that would not be captured by a “one service-by-one service” approach. The health status of an ecosystem needs the integrity of the ecosystem to be considered: this require holistic indicators to be applied.

To address policy questions concerning sustainable practices and degradation, it makes sense to focus on individual flows and to track back to their enabling actors (La Notte and Marques, 2017). This approach allows understanding who is accountable for what (i.e. to which economic sector to address policy action): a holistic approach may record that a change took place, but would not allow identifying the causes of this change (which ecosystem services involved) and their enabling actors.

The structure shown in Fig. 3, Fig. 5, Fig. 6 is consistent with the frame of external satellite accounts on which the SEEA-EEA is based and it is the structure adopted for the ongoing INCA applications for ecosystem services accounting in Europe for both supply and use tables and capacity accounts. As soon as applications will be made available, this structure will be tested, validated and eventually enhanced.

## Footnotes

1 For the most recent updates ref. https://seea.un.org/events/london-group-environmental-accounting-23rd-meeting.

2 Ref. https://www.wavespartnership.org/en/natural-capital-accounting.

3 Ref http://ec.europa.eu/environment/nature/capital_accounting/index_en.htm.

4 Ref. https://www.conservation.org/projects/Pages/Valuing-and-Accounting-for-Natural-Capital.aspx.

5 Ref http://www.teebweb.org/areas-of-work/advancing-natural-capital-accounting/.

6 Once available for all the ecosystem services provided by a specific ecosystem types, NPVs can be added up and eventually be calculated for ecosystem assets.
